# Use of cbct in the endodontic management of a second mandibular premolar with three roots: Clinical case

**DOI:** 10.4317/jced.58612

**Published:** 2022-01-01

**Authors:** Sebastiana Arroyo-Bote

**Affiliations:** 1 Associate professor. Faculty of Medicine and Health Sciences. Barcelona University. IDIBELL research. Coordinating teacher. Conservative Dentistry. ADEMA University School. University of the Balearic Islands. Spain

## Abstract

**Background:**

Reports and studies on dental anatomy describe variations in the morphology, number and shape of the roots and root canals, with the mandibular premolars being one of the dental groups with special complexity. In the bibliographic review published on the morphology of mandibular second premolars, the presence of three roots being extremely rare, finding differences according to the ethnic group studied. The aim of this case is to describe an unusual case of the anatomy of a mandibular second premolar.

**Material and Methods:**

A 59-year-old male patient presented with pain in the right mandibular second premolar. After taking the clinical history and the examination, he was diagnosed with irreversible serous pulpitis. Endodontic emergency treatment was started with the location of two root canals and the suspicion of two roots, according to radiographic examination. After performing CBCT, the presence of a third root was discovered in 45 and the presence of another anatomical variant in the right mandibular first premolar (44).

**Results:**

Endodontic treatment was performed in the three canals of 45 (mesio-vestibular, disto-vestibular and lingual). Clinical controls were carried out at 12 and 18 months.

**Conclusions:**

With the limitations of this clinical case, we can conclude that Anatomical knowledge is essential for performing endodontic treatment. The study of the tooth with CBCT is essential in cases where the anatomy of the tooth shows abnormalities in the radiographic study.

** Key words:**CBCT, Endodontic Management, Mandibular premolars, Radiography, Anatomy.

## Introduction

Reports and studies on dental anatomy describe variations in the morphology, number and shape of the roots and root canals, with the mandibular premolars being one of the dental groups with special complexity, possibly presenting more than one root (1,2). However, most mandibular premolars present a single root and a single root canal, although some studies have already reported the frequency of complex anatomies in the mandibular premolar canals, finding that 24% had two separate canals from the chamber pulp to the apex and 14% of the canals were C-shaped (3). The Vertucci classification on anatomical variables of root canals in mandibular premolars describes eight different types of anatomical configuration: Type I (1,1,1): a single canal from the pulp chamber to the apex ; Type II (2,2,1): two separate canals exit the pulp chamber joining near the apex to form a canal; Type III (1,2,1): a canal leaves the pulp chamber and divides in two at the root, which in turn merge to end as one; Type IV (2,2,2): two separate canals from the pulp chamber to the apex; Type V (1,1,2): one canal exits the pulp chamber and divides near the apex into two canals with separate apical foramina; Type VI (2,1,1): two separate canals exit the pulp chamber, merge in the root body and divide again near the apex to emerge as two distinct canals; Type VII (1,2,1,2): one canal exits the pulp chamber, divides and then rejoins in the root body, finally, it divides again into two different canals near the apex; Type VIII (3,3,3): three distinct and separate canals from the pulp chamber to the apex. Endodontic success fundamentally depends on a good diagnosis and an adequate strategy to access root canal treatment, so the anatomical knowledge of the tooth to be treated is very important for the planning and performance of root canal treatment.

Cone beam computed tomography (CBCT) is the most accurate complementary test for the study of the anatomy of the dental root and root canals *in vivo*; it is a method that offers us a large amount of information (4-8). In 2015, the American Association of Endodontists (AAE) and the American Academy of Oral and Maxillofacial Radiology (AAOMR) published the position on the use of CBCT in endodontics (9). Recently, the European Society of Endodontics in a position statement on the use of CBCT (5) stated that its use is indicated in patients with complex canal anatomy to obtain a good diagnosis and undertake endodontic treatment with knowledge of the root canal system that the tooth presents; however, its use should not be generalized in endodontic treatment.

We present the clinical case of a patient with complex anatomy in the right mandibular second premolar, in which CBCT helped identify the roots and root canals to perform root canal treatment.

## Case Report

This investigation complies with the Helsinki Declaration, and the patient signed an informed consent form.

A 59-year-old patient presented for spontaneous pain in the second right mandibular premolar (45), with two days of evolution, related to the fall of the obturation he had on the distal aspect.

The medical history detected no medical illnesses of interest. The dental history recorded a vertical dental fracture of 46, which required the extraction of the molar three months prior to the current clinical picture.

The patient explains having suffered severe and spontaneous pain radiating from the anterior teeth, which increases in reaction to thermal stimuli, presenting persistent pain.

**Figure 1:**
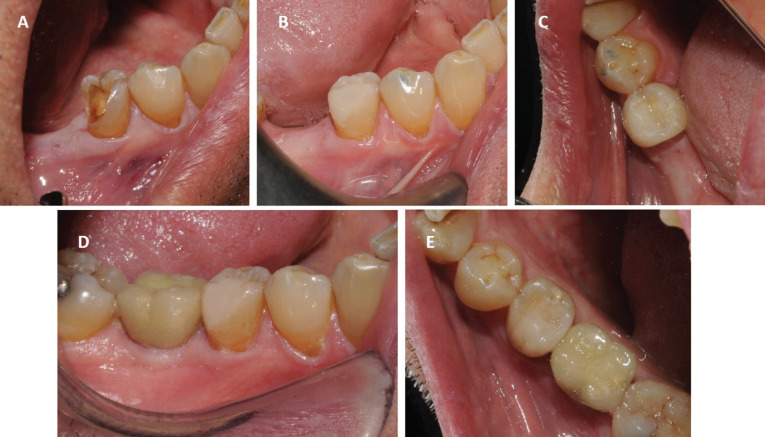
(A) Initial clinical image of the mandibular second premolar. Vestibular vision. (B) Clinical image at the end of the restoration. Vestibular vision. (C) Clinical image at the end of the restoration. Occlusal vision. (D) Clinical image one and a half years after restoration. Vestibular vision. (E) Clinical image one and a half years after restoration. Occlusal vision.

**Figure 2:**
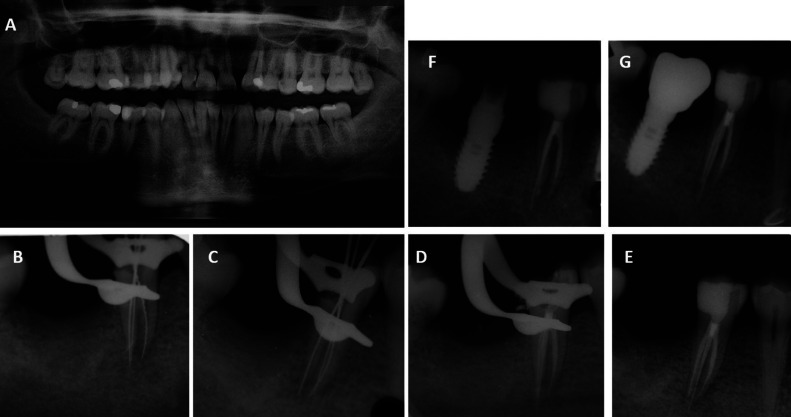
(A) Orthopantomography of the patient. (B). Periapical X-ray of conductometry on 45. (C) Periapical X-ray of conometry of 45. (D) Periapical X-ray at the end of endodontic treatment on 45. (E) Periapical X-ray at the end of restoration of the crown of 45. (F) Periapical X-ray one year after endodontic and restorative treatment of 45. (G) Periapical X-ray one and a half years after endodontic and restorative treatment of 45.

Examination revealed the absence of the disto-vestibular and occlusal wall of the right mandibular second premolar (45), with the presence of a cavity compatible with the loss of a restoration (Fig. 1-A). Palpation and percussion were not painful. The 45 vitality tests were increased, triggering severe pain. The 44 vitality test was normal. There was an oral orthopantomography performed three months before, where the distal obturation of 45 and the tooth anatomy were observed, identifying the presence of two roots (Fig. 2A).

Based on the clinical picture and the exploration, the patient was diagnosed with symptomatic irreversible pulpitis with no signs or symptoms of periapical pathology, and emergency endodontic treatment was indicated to relieve pain.

**Figure 3:**
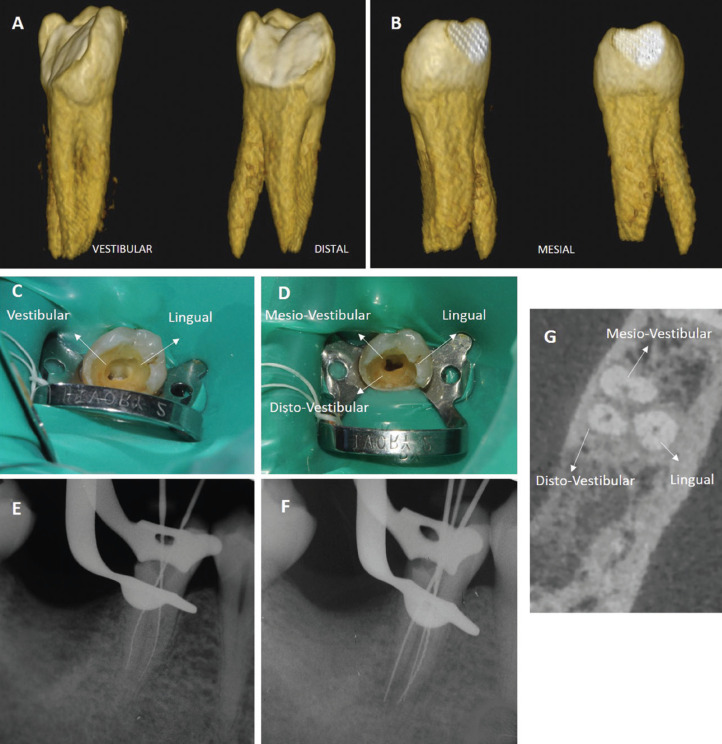
(A) Three-dimensional reproduction of the mandibular second premolar, seen from vestibular and distal. (B) Three-dimensional reproduction of the mandibular second premolar seen from mesial. (C) Cameral opening with the location of two canals. (D) Cameral opening with the location of three canals. (E) Periapical radiograph with the location of two roots and two canals. (F) Periapical radiograph with the location of the three canals and the three roots. (G) CBCT image with the three roots and the three canals.

After infiltrative anesthesia of 45 with SPC Xilonibsa 2% 1: 80,000 (Inibsa), the surgical field was isolated. Then, the cavity cleaning was started and the chamber was opened. Two canals, one vestibular and the other lingual, were located with K files 010-015 (Fig. 3C), with independent paths and starting on the buccal and lingual aspect of the pulp chamber. Several periapical X-rays were performed with different views (Fig. 3E). The radiographic study of the tooth can indicate the existence of anomalies and anatomical variants in the roots and root canals. After verifying that it was a tooth with a special anatomy, the root canal treatment was postponed, only removing the cameral pulp to relieve the patient’s pain.

The study of the tooth with CBCT is essential in cases where the anatomy of the tooth shows abnormalities in the radiographic study. In such a case, a CBCT scan is requested for anatomical study of the roots and root canals.

**Figure 4:**
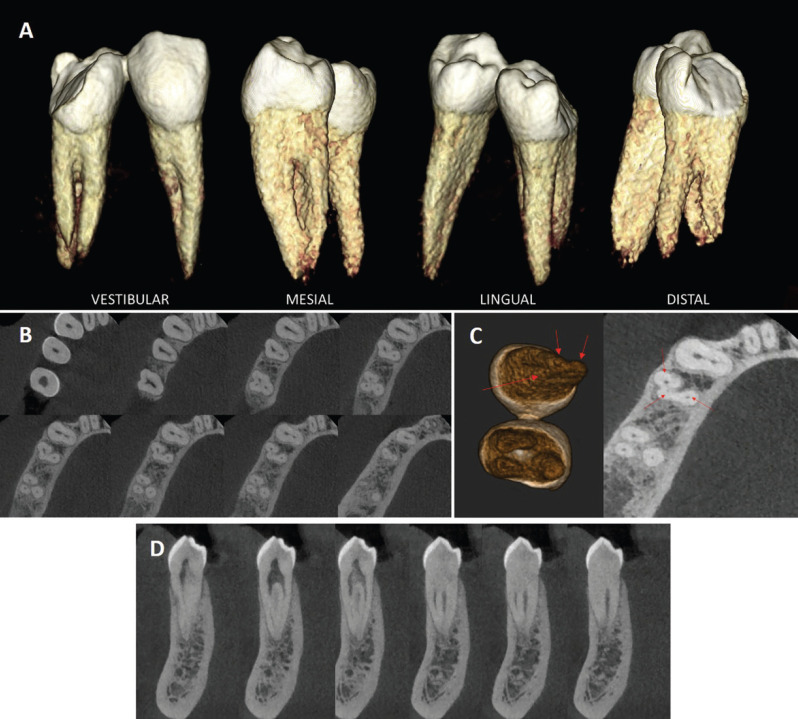
(A) Three-dimensional reproduction of the right mandibular first and second premolar. (B) Axial planes of 44 and 45 from pulp chamber to apex. (C) Three-dimensional reproduction of 44 and 45 seen from apical. Three foramina are seen at 44. In Axial section of 44 and 45. Three canals are seen at 44 and 45. (D) Coronal planes of 44 from distal face to mesial face.

The CBCT scan showed the presence of three roots, two located in the buccal and one in the lingual (Fig. 3A,B). The vestibular roots were of equal length, the lingual root was longer. Despite clearly differentiating, they were united in the coronal third, becoming independent in the middle and apical third where the lingual root was totally independent (Fig. 4B). Three independent circulating root canals were detected from the pulp chamber to the apex, one in each root (Fig. 3G). The canal anatomy was classified as Vertucci type I.

In the second appointment, infiltrative anesthesia and isolation of the operative field were performed again. The provisional filling was then removed, and the third canal was located. The three canals were: mesio-vestibular (MV), disto-vestibular (DV) and lingual (L) (Fig. 3D,F), as observed in the CBCT image (Fig. 3G).

Endodontic treatment was performed with a manual and rotary Protaper technique (Dentsply Maillefer, Ballaigues, Switzerland). We performed the Glide Path with K files - ISO 010 and 015. Little by little with the S1 rotary files with brushing movements and alternating with the file K015 we reached the working length in the three canals: L 19,5mm, MV 17mm and DV 16mm (periapical X-ray and apex locator). We instrumented with the S1 file until LT and later with S2. Then we proceeded to the apical conformation using F1 -F2 in the DV canal and F1-F2-F3 in the MV and L. The canals were permeabilized with a K015 file, after using each rotary file.

The canals were irrigated with sodium hypochlorite (NaOCl) to remove organic tissue and with 10% citric acid to dissolve inorganic matter.

5ml of 5.25% NaOCL were irrigated between each filing and in each canal with a 3ml syringe with a 30G side-vented irrigation needle (Monoject). The needle was inserted up to 2mm from the apex and slow irrigation was performed to avoid extruding irrigant to the periphery. The final irrigation was carried out with 10 ml of 5.25% NaOCL for 2 minutes, using passive ultrasonic irrigation with a 20 gauge-21 mm IrrisafeTM tip, followed by a wash with physiological serum. Then irrigation was done with 10% citric acid for 1 minute and after the wash we carried out the last application of 2% chlorhexidine for 1 minute. The canals were then dried with 25 and 30 gauge paper tips.

The obturation of the canals was performed with AHplus (Dentsply-Sirona) and gutta-percha cones of No. 25 in the DV, No. 30 canal in the MV and L, and lateral condensation tips.

Fig. 2B, C and D provide the periapical x-rays of the different phases of endodontic treatment.

In the same session, the crown was restored with aesthetic adhesive materials, using a self-etching adhesive with selective etching, a cavity base with fiberglass reinforcement and a micro-hybrid composite, and photographic control from the vestibular and occlusal areas at the end of the treatment (Fig. 1B,C) and periapical x-ray (Fig. 2E).

Months after the treatment of 45, an implant was performed in 46 (Fig. 2F) and after osseointegration the prosthetic crown was placed, which can be seen in the clinical and radiographic control of the restorative and endodontic treatment of 45, performed a year and a half after the root canal treatment (Fig. 1D,E, 2G).

## Discussion

The clinical case we present, a 45 with three roots and an independent root canal from the pulp chamber to the apex in each root, type I according to the Vertucci classification, is a very rare anatomical variant in mandibular second premolars. The arrangement of the roots and root canals is similar to the arrangement of the upper molars, recalling the molarization of the bicuspid, since there are two vestibular roots, disto-vestibular (DV) and lingual or buccal (L), and a buccal or lingual (L) root of greater length. Root development depends on the dynamic activity of the epithelial root lamina, although it may be under the stimulatory control of the dental papilla. The primary apical hole is subdivided into secondary apical holes, which is what causes the development of several roots, depending on the number of subdivisions that occur, that is the number of roots that will develop. Regarding the morphology of the crown despite the existing destruction due to the distobuccal cavity it presents, we can say that the dimensions in the vestibule-lingual and mesio-distal directions are normal.

In the bibliographic review published by Cleghorn BM *et al*. (10) on the morphology of mandibular second premolars, they concluded that 99.6% have a single root, two roots 0.3% and three roots only 0.1%, being extremely rare. Another systematic review carried out on mandibular premolars by Kottoor J, *et al*. (11) reported the presence of three or more canals in mandibular second premolars as scarcely being reported (0-2%), finding significant differences in the morphology of mandibular premolars according to the ethnic group studied. Alfawaz H *et al*. (12), in their study on the anatomy of mandibular premolars in the Saudi population, concluded that 95.6% of second premolars present type I canals, not finding any case of type VI and VII and finding the presence of type VIII in 1.7% and only three independent roots in 0.6% of the teeth with a significant difference between gender, being detected only in women, whereas the case reported here is from a male. Another study carried out on a Jordanian population (13) concluded that 72% of the second premolars studied have a single root and a single canal (Type I), and 22.8% had two roots with two canals and foramina separate apicals, not reporting any case of three roots. Another investigation carried out on the Nayarit population with CBCT (14) concluded that despite type I being the most frequent anatomical form (91.4%), the highest frequency of detected anatomical variables were types III and V, not finding any type VIII in the sample of 914 second premolars studied. Recent research carried out on the Thai population (15) did not find any cases of multiple roots in a total of 416 mandibular second premolars analyzed, detecting them in 5.73% of the 349 first premolars studied. The presence of anatomical variations is more frequent in the first premolar than in the second (2;11;12;15), which confirms the anatomical singularity of this clinical case, in which the most important anatomical anomaly is centered in 45.

Cleghorn BM *et al*. (16) published the clinical case of a patient with a first premolar with three roots and three canals and a second premolar with a C-shaped canal. The incidence of three roots in the mandibular first premolar is approximately 0.2%, being less frequent in the second premolar. Wong (17) reported endodontic success in a mandibular second premolar with four canals and a single root. Recently, Zhang M *et al*. (18) published a case of a mandibular first premolar with 5 canals, located thanks to the use of CBCT. In the case that we present, the CBCT allowed us to detect the third root that went unnoticed in the periapical radiographs, having three roots and three canals like the clinical case presented by Cleghorn BM in the first premolar. There are many studies that expose the different morphologies that the mandibular second premolar may present, which is why the clinician’s knowledge of the anatomy and the meticulous study of the tooth is necessary when planning endodontic treatment (19,20).

Regarding the anatomical symmetry with the contralateral premolar, Alfawaz H *et al*. (12) found a contralateral symmetry of 97.8% in terms of number of roots and canal conFiguration. Johnsen, GF *et al*. (21) also found symmetry in terms of number of roots and root canals but found variations at the apical level. However, previous studies (22) have concluded that relatively few pairs of contralateral teeth had anatomical symmetry. In this case, the anatomy of the contralateral premolar was not studied for ethical reasons; however, the CBCT study showed how the right mandibular first premolar also presented a rare anatomy (Fig. 4), which led us to suspect, together with the orthopantomography (Fig. 3-A), that the contralateral premolars also had more than one root or root canal. In Fig. 4-A we observe the 3D reproduction of the two premolars (44-45), appreciating the spatial arrangement of the roots. In Fig. 4B,D the most representative sections of the CBCT study are shown (Fig. 4B axial planes and Fig. 4D coronal planes). In 44, there is the division of two clearly differentiated roots and a third distal formation of the vestibular root that remains fused to both roots, but that shares a canal with the vestibular root, separating in the final part with an independent foramen (Fig. 4C) from the apical foramen of the vestibular root (Fig. 4C). Therefore, the vestibular root of 44 would be classified as Vertucci type V and the lingual as type I. The vestibular root in some axial planes (4B) suggests a configuration in C such as those described by Jan *et al*. (2) and Ordinola-Zapata *et al*. (23). In Figure 4C the three foramina were confirmed, two in the buccal root and one in the lingual root, by 3D reproduction and an axial section.

## Conclusions

With the limitation of this manuscript, it can be concluded that we must perform a good radiographic examination when faced with endodontic treatment of mandibular premolars to detect anatomical variations. The study with CBCT is necessary to obtain good knowledge of the anatomy and morphology of the roots and root canals whenever some type of dental anomaly is suspected.

## References

[1] Zoya-Farook A, Abhishek P, Shahabadi A (2017). Cone-beam Computed Tomographic Evaluation and Endodontic Management of a Mandibular First Premolar with Type IX Canal Configuration: Case Report. J Endod.

[2] Jang YE, Kim Y, Kim B, Kim SY, Kim HJ (2019). Frequency of non-single canals in mandibular premolars and correlations with other anatomical variants: an in vivo cone beam computed tomography study. BMC Oral Health.

[3] Baisden MK, Kulild JC, Weller RN (1992). Root canal configuration of the mandibular first premolar. J Endod.

[4] Ok E, Altunsoy M, Nur BG, Aglarci OS, Çolak M, Güngör E (2014). A cone-beam computed tomography study of root canal morphology of maxillary and mandibular premolars in a Turkish population. Acta Odontol Scand.

[5] Patel S, Durack C, Abella F, Shemesh H, Roig M, Lemberg K (2015). Cone beam computed tomography in Endodontics - a review. Int Endod J.

[6] Ordinola-Zapata R, Bramante CM, Versiani MA, Moldauer BI, Topham G, Gutmann JL (2017). Comparative accuracy of the Clearing Technique, CBCT and Micro-CT methods in studying the mesial root canal configuration of mandibular first molars. Int Endod J.

[7] Piasecki L, José Dos Reis P, Jussiani EI, Andrello AC (2018). A Micro-computed Tomographic Evaluation of the Accuracy of 3 Electronic Apex Locators in Curved Canals of Mandibular Molars. J Endod.

[8] Alamri HM, Mubashir B, Mirza Alharbi F, Aljarbou F (2020). Root canal morphology of maxillary second molars in a Saudi sub-population: A cone beam computed tomography study. Saudi Dent J.

[9] Special Committee to Revise the Joint AAE/AAOMR Position Statement on use of CBCT in Endodontics (2015). AAE and AAOMR Joint Position Statement: Use of Cone Beam Computed Tomography in Endodontics 2015 Update. Oral Surg Oral Med Oral Pathol Oral Radiol.

[10] Patel S, Brown J, Semper M, Abella F, Mannocci F (2019). European Society of Endodontology position statement: Use of cone beam computed tomography in Endodontics: European Society of Endodontology (ESE) developed by. Int Endod J.

[11] Cleghorn BM, Christie WH, Dong CC (2007). The root and root canal morphology of the human mandibular second premolar: a literature review. J Endod.

[12] Kottoor J, Albuquerque D, Velmurugan N, Kuruvilla J (2013). Root anatomy and root canal configuration of human permanent mandibular premolars: a systematic review. Anat Res Int.

[13] Alfawaz H, Alqedairi A, Al-Dahman YH, Al-Jebaly AS, Alnassar FA, Alsubait S (2019). Evaluation of root canal morphology of mandibular premolars in a Saudi population using cone beam computed tomography: A retrospective study. Saudi Dent J.

[14] Awawdeh LA, Al-Qudah AA (2008). Root form and canal morphology of mandibular premolars in a Jordanian population. Int Endod J.

[15] Barrón-Plata AL, Sánchez-Huerta EA (2019). Identificación de variables en la morfología de conductos en primeros y segundos premolares mandibulares, mediante tomografía computarizada Cone Beam en población nayarita. Oral.

[16] Arayasantiparb R, Banomyong D (2021). Prevalence and morphology of multiple roots, root canals and C-shaped canals in mandibular premolars from cone-beam computed tomography images in a Thai population. J Dent Sci.

[17] Cleghorn BM, Christie WH, Dong CC (2008). Anomalous mandibular premolars: a mandibular first premolar with three roots and a mandibular second premolar with a C-shaped canal system. Int Endod J.

[18] Wong M (1991). Four root canals in a mandibular second premolar. J Endod.

[19] Ordinola-Zapata R, Bramante CM, Cavenago BC, Duarte MH, Versiani MA (2013). Morphologic micro-computed tomography analysis of mandibular premolars with three root canals. J Endod.

[20] Kharouf N, Haikel Y, Mancino D (2019). Root Anatomy of Mandibular Second Premolars in French Subpopulation: A Retrospective Observational Case Series. Contemp Clin Dent.

[21] Johnsen GF, Dara S, Asjad S, Sunde PT, Haugen HJ (2017). Anatomic Comparison of Contralateral Premolars. J Endod.

[22] Xu J, Shao MY, Pan HY, Lei L, Liu T, Cheng L (2016). A proposal for using contralateral teeth to provide well-balanced experimental groups for endodontic studies. Int Endod J.

[23] Ordinola-Zapata R, Monteiro Bramante C, Gagliardi Minotti P, Cavalini Cavenago B, Gutmann JL, Moldauer BI (2015). Micro-CT evaluation of C-shaped mandibular first premolars in a Brazilian subpopulation. Int Endod J.

